# Extraction Socket Augmentation with Autologous Platelet-Rich Fibrin (PRF): The Rationale for Socket Augmentation

**DOI:** 10.3390/dj11080196

**Published:** 2023-08-14

**Authors:** Cemal Ucer, Rabia S. Khan

**Affiliations:** I.C.E Postgraduate Dental Institute and Hospital, University of Salford, Salford M5 4WT, UK; ucer@icedental.institute

**Keywords:** platelet-rich fibrin, ridge augmentation, socket augmentation, grafting, cytokines, growth factors, tissue regeneration, bio-enhancement, PRF, osteogenesis

## Abstract

After tooth extraction, the alveolar ridge undergoes a physiological process of remodelling and disuse atrophy. Socket augmentation (SA) has been shown to preserve alveolar bone volume in order to facilitate implant placement and reduce the need for staged grafting at a later date. Although autogenic grafting has been shown to be the gold standard in bone regeneration, it has significant disadvantages. To prevent post-extraction volumetric alterations and alveolar bone resorption occurring, alternative grafting materials, including xenografts, alloplasts, and allografts, have been used successfully in fresh extraction sites. However, these materials act mostly as bio-scaffolds and require a slower integration period of 6–8 months prior to implant placement. Recently, the use of autologous platelet-rich fibrin (PRF) has been advocated alongside socket augmentation as a method of bio-enhancement of healing of soft and hard tissues. PRF contains platelet-derived growth factors, hormones, and bioactive components such as cytokines that have been shown to promote angiogenesis and tissue regeneration during wound healing. The aim of this article is to review the evidence base for the SA technique Clinical benefits of SA will be discussed with a reference to two cases. Therefore, this narrative review will discuss the post-extraction bone changes, the importance of SA, and the bio-enhancement role of PRF in the management of extraction site defects when the alternative technique of immediate implant placement is not possible or contraindicated.

## 1. Introduction

Dental implants have become an integral part of dentistry for the replacement of failing teeth and their supporting tissues to restore dental function and aesthetics. More recently, immediate, or delayed implant placements in fresh extraction sites have been advocated to manage tooth loss. To achieve a successful long-term outcome with dental implants, there are certain anatomical, aesthetic, functional, and biomechanical prerequisites. Of particular importance for the integration process, as well as the long-term stability of peri-implant tissues, is the quality and quantity of the hard and soft tissues available at the recipient site. When a tooth is lost, the alveolar bone goes through a process of physiological remodelling that results in specific anatomical changes in the quality and quantity of the bone surrounding the root socket. This was first reported by Johnson (1969) [1]. The bundle bone, facing the root surface within the socket, resorbs rapidly first in response to loss of a tooth [2]. The alveolar bone undergoes a slower process of volumetric changes thereafter.

We applied the methodology of a narrative review, including randomised clinical trials (RCT) with case reports, as a suitable understanding of the mechanism of socket healing is essential for the decision-making process when managing a tooth replacement, especially with reference to the timing of the different stages of implant therapy [2,3].

### 1.1. Post-Extraction Alveolar Bone Resorption

Alveolar bone resorption at the tooth socket begins horizontally, resulting in the loss of the buccal wall of the socket at first. The subsequent reduction in socket wall height causes three-dimensional (3D) alterations in socket morphology, resulting in a narrower ridge profile with reduced height. When the pre-extraction labial bone is thin (e.g., <2 mm), changes in socket morphology become particularly more pronounced.

Maia et al., 2016 reported a higher bone loss in extraction sites with a thin gingival biotype or thin buccal bone plate [4]. As a result of remodelling, the post-extraction prosthodontic trajectory of the alveolar ridge shifts from the buccal to the lingual plane. This creates a defective residual ridge anatomy that often complicates the ideal placement of an implant, thus necessitating a simultaneous or even staged grafting procedure to regenerate the lost alveolar bone volume. It has been reported that 50% of all implants, as well as almost 75% of implants placed in the anterior maxilla, require bone augmentation [5]. This increases the complexity, cost, as well as the overall duration of implant rehabilitation.

The clinical decision process on how best to manage the transition from a tooth requiring extraction to its replacement with an implant has been addressed in numerous systematic reviews (SRs) and consensus conference reports. The options include immediate/early implant placement or delayed implant placements either after socket augmentation or unaided socket healing [6]. The conventional treatment protocol has involved tooth extraction and unassisted healing of the extraction site for a period of >16 weeks, followed by implant placement in a healed ridge albeit after significant alterations to alveolar bone morphology had taken place. A modified protocol involves treatment of the extraction socket to minimise the dimensional changes that take place after tooth extraction, followed by implant placement 12–16 weeks later. This approach has been called alveolar ridge preservation (ARP) [7]. The XV European Workshop in Periodontology (2019) consensus meeting has concluded that there is now a substantial and expanding evidence base available to guide clinicians in clinical decision-making process when managing the extraction socket and timing of implant placement [8].

#### 1.1.1. Stages of Post-Extraction Changes

Post-extraction alveolar bone atrophy occurs at a rate of 50–60% during the first three months after tooth loss and continues indefinitely thereafter, although at a much lower rate [9,10,11,12]. Periodontal soft tissue atrophy gradually mirrors alveolar bone loss in an apical direction. These dimensional changes to bone and soft tissues have a profound impact on the prosthodontically guided delivery of implant treatment and negatively affect its outcome with respect to functional, aesthetic, and anatomical aspects of the case and patient expectations.

Post extraction dimensional changes occur primarily due to disuse atrophy that starts with the loss of bundle bone during the first 7–14 days post-extraction. The physiological host response is to reduce the width and height of the walls of the extraction socket in order to seal the soft tissue defect as quickly as possible with oral mucosa. In addition to the physiological response of disuse bone remodelling, bone loss could be pronounced due excessive surgical trauma, presence of root ankylosis, post-extraction infection, smoking, ill-fitting prostheses, as well as reduced blood supply due to soft tissue trauma or lifting of periosteum. Thin labial plate could be inadvertently lost when using a conventional tooth extraction technique. Traumatic tooth loss could also contribute to substantial bone loss, particularly in the anterior maxilla, where the entire thin labial bone could be lost due to fracture. These volumetric changes to alveolar ridge morphology compromise the optimum placement of dental implants and could increase the complexity of treatment by necessitating staged hard and soft tissue grafting procedures, thus increasing the overall duration and cost of implant rehabilitation [13,14,15,16,17]. When the buccal wall is 1 mm or narrower after tooth extraction, a median vertical bone loss of 7.5 mm of the socket buccal wall could be expected at eight weeks post-extraction (Chappuis et al.). This has implications for implant rehabilitation, especially in the pre-maxillary area, where the buccal bone thickness has been shown to be less than 1 mm in 90% of cases [18]. Ortiz G et al. have recommended that SA would be particularly beneficial in extraction sites with thin walls to preserve the alveolar ridge before implant placement [19].

#### 1.1.2. Post-Extraction Socket Augmentation

While the efficiency of SA in preventing gross dimensional changes over unassisted socket healing has been demonstrated, both clinically and histomorphometrically, in randomized controlled trials and systematic reviews, some authors have questioned the cost/clinical effectiveness of this technique, citing a large variation in previously reported results. They have further argued that some studies have revealed only marginally better horizontal bone preservation of about 1 mm in SA sites compared with untreated extraction sockets, and this would not be of clinically significance. Another objection has been the slow conversion of xenograft bone graft materials and a possible detrimental effect of the relatively high percentage of remaining graft material on the osseointegration process [19]. There have also been concerns regarding the stability of marginal bone around implants placed in grafted sockets.

Although SA has been shown to be effective in limiting horizontal and vertical ridge resorption following tooth extraction, it should be noted that some bone loss will still occur, and complete preservation of pre-extraction socket dimensions may not always be possible (Mardas et al.) [20]. This is particularly relevant given the variability of the biological age of subjects, the type of extraction technique, the site of extraction, surgical trauma, morphology and size of the extraction socket and its wall thickness, and different biological properties of various graft materials used in different studies. To add to this variability, numerous SA techniques have been proposed, such as open vs. fully flapped healing with or without the use of barrier membranes. Nevertheless, even a minimum amount of millimetric bone preservation would clinically be beneficial when compared with several millimetres of bone loss that occurs typically after unaided socket healing.

The optimum time for socket healing after SA is not known. In most SA studies, a healing period of 6–7 months has been allowed before the placement of implants. It is likely that this extended period of healing, in the absence of functional loading, would have contributed to additional bone remodelling and a further reduction in socket dimensions. This observation is supported by good-quality RCT and CCTs illustrating the bioactive role of PRF in tissue regeneration in SA. Nevertheless, the optimum healing period and the role of PRF in enhancing bone graft healing during SA need to be investigated in future studies.

Another factor that influences the level of bone loss would be the surgical trauma caused to the periosteum and the blood supply. A full-thickness muco-periosteal flap is normally raised to facilitate the placement of a barrier membrane or to achieve primary closure of the extraction site. This may have resulted in additional loss of bone around extraction sockets seen in some studies that have employed a primary closure technique with periosteal flaps. Mardas et al. (2010) have suggested that in cases of SA, flapless techniques should be utilised, to prevent further crestal bone resorption occurring due to damage to the periosteum and the blood flow. The benefit of flapless SA should therefore be investigated urgently to see if this would affect the results [21].

De Risi V et al. [22] carried out an SR of the histological and histomorphometrical data of different biomaterials used for alveolar ridge preservation procedures on healing after tooth extraction in humans. The percentages of new bone produced at 3 months varied, with allografts showing 54.4% new bone, while the lowest was obtained, at 5 months, with xenografts (23.6%). The lowest rates of residual bone grafts were displayed by allografts (12.4–21.11%), while those sites using xenografts and alloplasts showed the best results at 7 months 37.14 and 37.23%, respectively [22].

### 1.2. Decision Tree for timing of Implant Placement

Socket augmentation or immediate/early implant placement are two treatment modalities that have been shown to be equally effective in mitigating against post-extraction anatomical changes and improving patient outcomes [23,24]. The technique of immediate or early implant placement in fresh extraction sites has been demonstrated, in numerous studies, to reduce the unwanted dimensional changes to the hard and soft tissues surrounding an extraction socket while ensuring suitable aesthetic outcomes and long-term success rates [25,26,27].

In a recent randomized clinical trial (RCT), Jonker et al. [28] investigated early implant placements in extraction sockets treated with or without alveolar ridge preservation (SA) and showed that both protocols had resulted in suitable aesthetic and clinical outcomes due to the prevention of post-extraction hard and soft tissue dimensional changes. Thus, concluded that there was negligible value in carrying out a staged SA procedure if early implant placement can be undertaken within 8 weeks of tooth removal. The authors, however, pointed out that the protocol of early implant placement at 8 weeks would have taken place before most of the hard tissue alterations had occurred and that the shorter healing period may not have been sufficient for proper consolidation of SA, thus hampering its benefits. Nevertheless, a great majority of early implant placements undertaken in untreated extraction sites (non-SA) had required a simultaneous GBR procedure to treat labial bone defects (<2 mm buccal bone thickness) compared with SA-treated extraction sites. This finding is consistent with recent studies that have shown that the technique of socket augmentation (SA) followed by early implant placement reduces the frequency of simultaneous GBR at early implant placement, thus simplifying the surgical procedure [28].

Immediate implant placement is not always possible or ideal when there is substantial volume of labial socket wall missing or when primary implant stability is not achievable. The presence or absence of an intact residual ridge or damaged socket walls are normally used as decision-making criteria when selecting a specific technique. When immediate implant placement is not possible or contraindicated, the technique of “ridge augmentation” (RA), also referred to as “socket augmentation” (SA) has been established as an evidence-based standard treatment option when planning tooth replacements after extractions [28]. Table 1 summarises the strategies for management of tooth loss with dental implants and factors that should be considered when making a decision regarding the management of extraction sockets and timing of implant placements.

Table 2 presents a summary of the factors that can impact the clinical decision making process when considering the timing of implant placement and SA.

These factors have been adapted from the XV European Workshop in Periodontology Consensus, providing a comprehensive overview of the key considerations that dental professionals need to take into account when making clinical decisions in managing tooth loss with dental implants.

### 1.3. Extraction Socket Healing and Dimensional Changes

**Figure 1:**
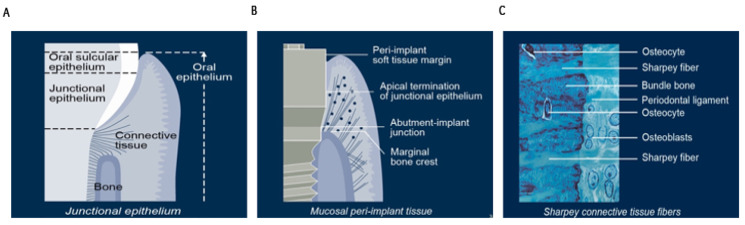
Post-extraction physiological alveolar ridge remodelling and disuse atrophy. (**A**) After tooth loss, soft tissues lose their attachment to the cementum layer of the root surface. (**B**) The bundle bone resorbs once the Sharpey’s fibre attachments are lost. (**C**) As the interdental and crestal bone resorb, the gingival tissues recede in an apical direction. These volumetric changes reduce the available bone volume for implant anchorage and adversely affect the future aesthetic scores of the case, which are based on the height of the interdental papilla, gingival margin, and clinical crown height (printed with permission of ITI).

Dimensional bone and soft tissue changes following tooth extraction have a significant negative impact on tooth replacement, particularly in the aesthetic zone. The bone and soft tissues recede in an apical direction with functional, anatomical, and aesthetic consequences. These changes have an adverse effect on the primary stability of the implant and subsequent maintenance of osseointegration. Moreover, loss of bone support has a profound long-term influence on the quality and biotype of the overlying peri-implant soft tissues (Figure 1) [29,30].

### 1.4. Stages of Extraction Socket Healing

#### 1.4.1. Haemostasias and Inflammation Phase

Haemostasis occurs in response to injury. Acute inflammation is the first reaction of the immune system to injury, which is mediated through a cellular response involving platelets, leukocytes, and macrophages. Platelets start aggregating and become activated in contact with collagen. Thrombin is involved in the fabrication of fibrin mesh during this phase of wound healing. During this stage, the host tissues try to eliminate bacteria and cell debris. Initially, polymorphonuclear neutrophils enter the wound, followed by macrophages that differentiate from monocytes. Monocytes are produced in the bone marrow. Monocytes differentiate into macrophages in injured tissue. They are also activated by T-lymphocytes as part of the osteoimmune response, in which cells from the immune and skeletal systems share the same microenvironment and interact extensively through various cytokines and signalling pathways. Macrophages, apart from phagocytosis, produce growth factors that enhance angiogenesis, granulation tissue formation collagen synthesis [31]. This inflammatory stage is a prerequisite for subsequent stages of proliferation and tissue regeneration and mineralisation.

#### 1.4.2. Cell Proliferation Phase

The proliferative phase begins with the formation of a fibrin, fibronectin glycosaminoglycan, and hyaluronic acid matrix that is initially populated with macrophages and platelets. The various cytokines secreted by these cells enhance cell migration into the site using the fibrin and fibronectin matrix as a scaffold. Progenitor cells are recruited from bone, cartilage, muscle, nerve sheath, and connective tissue cells [32]. Physiologically, platelets are the primary component of haemostasia in response to tissue injury. They form a scaffold of the fibrin network [33]. After initial haemostasis, through the release of growth factors and cytokines, platelets also play a key role in the subsequent stages of wound healing (e.g., haemostasias, inflammation, proliferation, maturation phases) and bone regeneration by chemotaxis, cellular recruitment and proliferation, and gene expression in fibroblasts and macrophages.

Cytokines, a subtype of growth factors that are produced by platelets and other haematopoietic and immune cell types, (e.g., interferons and interleukins) also promote cellular differentiation and cell division [34,35]. During the proliferation phase angiogenesis, collagen and granulation tissue formation followed by wound contraction and epithelization occur under influence of the above-mentioned bioactive substances.

New tissue formation occurs during the first 10 days after injury and is characterised by cellular recruitment, proliferation, and the migration of different cell types. New blood vessels are formed by a process known as angiogenesis. The osteoimmunological process involves several signalling pathways and cell-to-cell communications mediated by growth factors, including platelet-derived growth factor (PDGF), vascular endothelial growth factor (VEGF), and cytokines. Fibroblasts and macrophages replace the fibrin matrix with granulation tissue during the maturation phase of wound healing [36,37,38,39]

Platelets and leukocytes, therefore, play a crucial role in all stages of wound healing, from coagulation to angiogenesis and activation of cells (e.g., monocytes, neutrophils, fibroblasts macrophages, and mesenchymal stem cells) that are involved in tissue regeneration (Table 3).

## 2. Indications for Socket Augmentation

The rationale for SA is to allow full bone regeneration within the extraction socket to facilitate prosthodontically guided 3D-implant placement at later stages. Although the optimum timing of implant placement after SA is not known [47,48], there is a consensus that implant placement should be deferred for up to a period of 16–24 weeks after tooth removal. Such an extended period of healing is thought to be necessary to allow for initial graft conversion and bone maturation (Kalsi et al., 2019) [49]. It is important to recognize also that studies have demonstrated a wide range of remodelling properties and characteristics between different types of bone substitute materials. Some biomaterials resorb and incorporate within new bone faster (e.g., allografts) than others. To reduce the prolonged healing time, enhancement of the healing process in conjunction with SA has been advocated with the use of bioactive substances such as hyaluronic acid (HA), collagen matrix, bone morphogenic proteins (BMPs), or platelet-rich fibrin (PRF) [50].

### 2.1. Indications for Socket Augmentation at the Time of Tooth Loss

- To preserve and augment hard and soft tissues to mitigate against post-extraction socket remodelling when future implant placement is being planned, see Table 4;
- To optimise the future implant site for prosthodontically guided, 3D placement of an implant to fulfil aesthetic, functional, and biomechanical requirements and patient expectations;
- To regenerate bone within the socket to facilitate future implant placement with improved primary stability;
- To prevent gross post-extraction alveolar ridge reduction in sites:
  ◦ With damaged socket walls;
  ◦ With thin gingival biotype or thin buccal wall thickness <2 mm;
  ◦ In close proximity to apically related anatomical structures such as the maxillary sinus or inferior alveolar nerve.

### 2.2. Case Study

**Figure 2:**
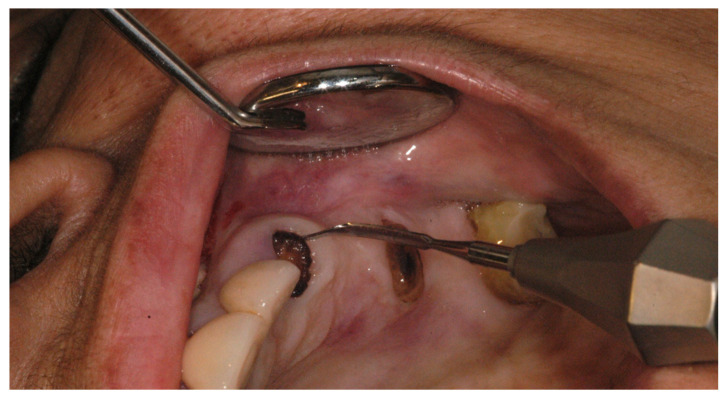
Minimally invasive tooth extraction technique using special instruments such as a periotome (as seen in this image) or piezo surgery (with extraction inserts) is recommended to avoid trauma to the thin sockets of the extraction socket.

**Figure 3:**
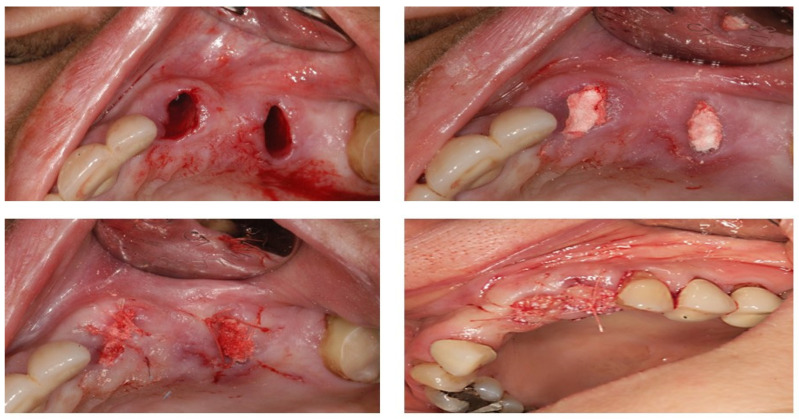
(Minimally invasive tooth removal was carried out (surgeon: T. C. Ucer).

Figure 2 shows the use of periotome for the extraction of roots. Whereas, Figure 3 explains that after infiltration with a local anaesthetic solution, the socket was debrided of all remnants of granulation tissue of any periapical pathology and irrigated with saline. (The socket was augmented with a synthetic graft (Ethoss^®^), which can be left to heal with or without a covering of a membrane, a connective tissue graft, or PRF fibrin in a *semi-open* healing technique with no attempt to close the extraction site defect primarily. (c) Mattress sutures were used to keep the edges of the wound intact. In this case, socket augmentation was realised using a novel synthetic biomaterial consisting of a composite mixture of 65% beta-tricalcium phosphate and 35% calcium sulphate that allows in situ hardening. No primary soft closure or use of a barrier membrane is necessary with this material (Ethoss^®^).

**Figure 4:**
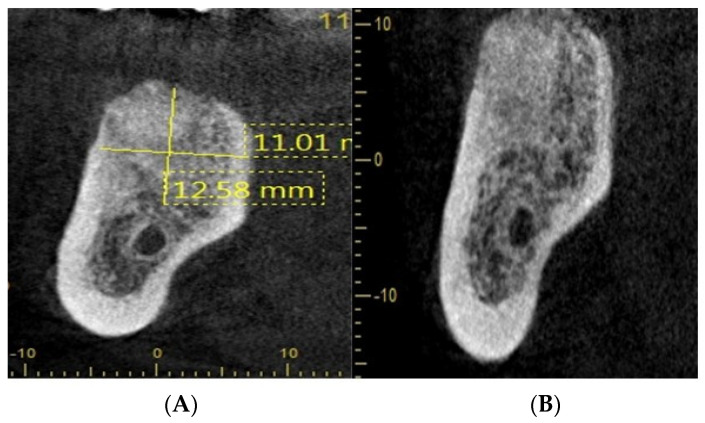
(**A**,**B**) The aim of SA is to reduce the dimensional changes occurring to the extraction socket/alveolar bone profile after tooth removal and to regenerate bone for optimum implant placement at a later date.

Figure 4 shows radiographs showing suitable bone density at 8 weeks after socket augmentation of mandibular molar teeth carried out at the time of tooth extraction. Note the general maintenance of the alveolar ridge shape and volume after SA. The rate of bone graft remodelling/conversion has been shown to be faster with allografts/xenografts compared with a xenograft. There is strong accumulating evidence that the quality and quantity of bone regeneration can be accelerated when PRF is used in conjunction with SA (surgeon: T. C. Ucer).

**Figure 5:**
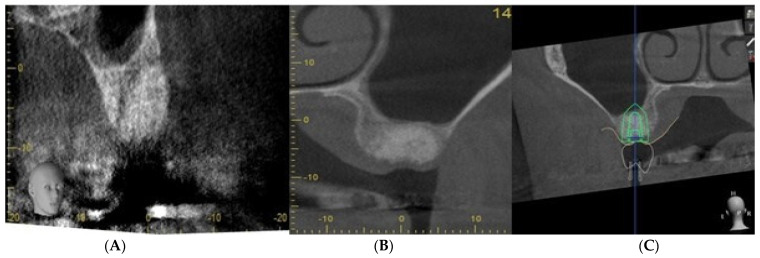
CBCT sections, (**A**–**C**) showing ridge preservation after SA.

Figure 5 illustrates that socket augmentation can prevent further pneumatisation of the maxillary sinus floor and maintains the socket volume, thus allowing straightforward placement of an implant below the sinus floor without any need for crestal sinus lift or lateral window sinus floor grafting. Section C shows the planning of a short implant (length: 7 mm) after SA of the UL 6 extraction socket (surgeon: T. C. Ucer).

**Figure 6:**
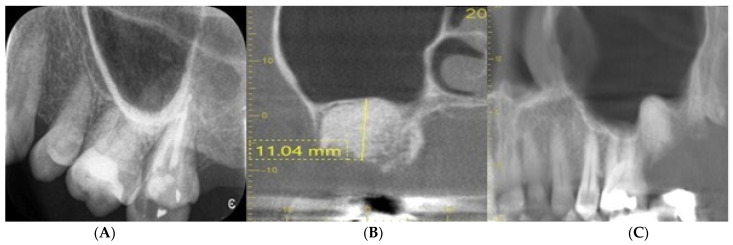
CBCT sections (**A**) shows the upper left molars before extraction (**B**,**C**) showing preservation of the alveolar ridge morphology following SA of UL 7 extraction site.

Figure 6 shows the periapical radiograph (Image A) shows the pre-extraction condition of the UL7 molar tooth, which had horizontal marginal bone loss in very close proximity of the maxillary sinus floor. On extraction of UL7, the socket was debrided carefully of all remnants of granulation tissue and augmented with BioOss^®^. The socket was allowed to heal in a semi-open condition with 3.0 Vicryl^®^ matress suture covering the soft tissue defect without no attempt to achieve primary closure. Follow-up CBCT images (Image B and C) show the healed grafted socket filled with dense radiopaque bone material with at least 11 mm residual bone remaining below the maxillary sinus floor (surgeon: T. C. Ucer).

One of the benefits of SA is to avoid socket collapse when planning the implant replacement of a molar tooth with a reduced alveolar bone height below the floor of the maxillary sinus. The above radiographs in Figure 5 and Figure 6 illustrate the concept of alveolar bone preservation (SA) when extracting a molar tooth in close proximity to the maxillary sinus cavity that allows the placement of a short implant at a later date, thus avoiding more invasive sinus floor elevation.

**Figure 7:**
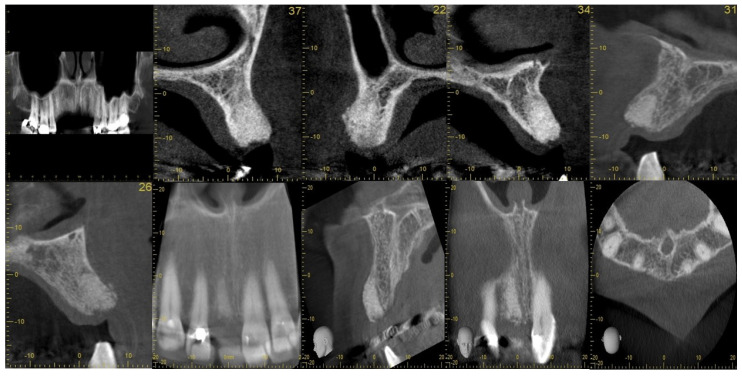
Socket augmentation in the aesthetic zone to preserve the alveolar ridge for staged implantation and to achieve optimum aesthetic results. The aim is to prevent the resorption of the thin labial plate of the socket.

Figure 7 shows SA and particularly indicates the case when the labial socket wall is missing or is <1 mm in thickness.

SA is particularly indicated when primary immediate implant stability cannot be obtained in an extraction site due to the presence of an insufficient amount of bone (horizontally, vertically, or both). This commonly occurs in a large cystic defect or when the residual bone height is limited due to apical proximity of the floor of the maxillary sinus or a neurovascular bundle. SA is also indicated when immediate implant placement is not planned or when the tooth extraction results in a large defect of unfavourable morphology (e.g., a large molar tooth socket), where ideal positioning of an implant would not be possible. In these cases, the purpose of SA is to provide a bio-scaffold for optimum hard and soft tissue healing to facilitate best implant positioning at a later stage as suggested by Kim et al. [51]. Socket augmentation can be carried out in stages or simultaneously at the time of immediate implantation using a simple socket seal technique with a barrier membrane or in conjunction with a full GBR procedure with or without primary closure.

Several variations of the SA technique have been reported in the literature in controlled studies using various bone grafting materials, including autografts, allografts, xenografts, and alloplasts. Flapless SA, semi-open healing with or without the use of barrier membranes or collagen sponges have been advocated, with suitable results reported for these different approaches. There is, however, no consensus as to the optimum technique or choice of a biomaterial when carrying out SA. More recently, PRF has been used to enhance tissue regeneration in conjunction with SA [52,53,54,55,56,57,58,59,60,61,62].

### 2.3. Evidence for Socket Augmentation

There have been numerous publications demonstrating the effectiveness of socket augmentation (SA) in recent years. Given the wide variety of techniques and biomaterials used with very little standardisation, inevitably, inconsistent results have been reported on the clinical benefits of socket augmentation for the prevention of the post-extraction socket wall resorption Jung et al. (2004) and Nevins et al. (2006). It has often been impossible to compare these results due to the heterogeneity of outcome measures and study designs used in these studies. Moreover, some researchers have objected to the concept of SA, arguing that socket grafting could be detrimental to osseointegration, as the graft material could impair or slow down the natural healing process and formation of new bone (Becker et al., 1998; Araujo and Lindhe 2009; Araujo et al., 2009) [63,64,65].

Adams (2022), based on a subjective review of some SRs, has reported that, despite several systematic review and meta-analysis studies demonstrating a significant reduction in socket dimensional changes following alveolar ridge augmentation (RA) compared with extraction alone, there was a low level of evidence to support alveolar ridge preservation and that the utility of this technique must therefore be questioned as the treatment of choice for the management of extraction sites. The author further cited a high-risk SR that showed a mean reduction in dimensional changes after RA of 1.18 mm horizontally and 1.35 mm vertically and declared that the clinical benefit of such a small amount of bone preservation was unproven and unwarranted [66]. On the other hand, numerous studies have shown that even a small amount of bone preservation (e.g., 1 mm), particularly in the aesthetic zone, would be highly preferable.

### 2.4. Clinical, Radiological, and Histological Investigations Validating the Technique of Socket Augmentation

There is growing consensus that a suitable level of evidence for SA has now been made available, with the publication of numerous controlled studies and systematic reviews, showing that extraction socket augmentation with a biomaterial with or without a barrier membrane reduces the degree of dimensional changes that occur due to disuse atrophy.

In a CT scan investigation, studied the physiological phenomenon of post-extraction alveolar bone resorption and compared it with fresh extraction sites treated with a xenograft (Bio-Oss^®^) covered with a coronally advanced flap. They reported that this technique of SA provided a significant benefit in maintaining the original bone volume in treated sites [67]. Nevertheless, the histologic data showed that the residual graft conversion process was slow and was still ongoing even after 7 months, with this choice of biomaterial demonstrating again that xenografts are thoroughly a scaffold material with very slow turnover properties.

Several studies, including that by Araujo et al., have reported better crest maintenance using graft materials in fresh extraction sites. Further evidence of the longitudinal stability of flapless socket augmentation (SA) was provided by two randomised controlled clinical studies (Cornelini et al., 2004 and Chen et al., 2007) in which the technique of socket augmentation with and without membranes was demonstrated to maintain buccal bone volume at extraction sockets [68,69]. Kalsi et al. have reported alveolar ridge augmentation (RA) to be a predictable procedure to reduce undesirable horizontal and vertical ridge reduction following tooth extraction and proposed an evidence-based protocol for decision making when choosing between immediate implant placement and SA at the time of tooth loss [70].

A randomised controlled trial conducted by Sisti et al. (2012) showed radiographically near complete vertical and horizontal maintenance of the grafted volume with flapless socket augmentation. They further showed that the application of particulate socket grafts without barrier membranes minimised alveolar crest resorption in large fresh extraction sites and resulted in better horizontal regeneration of the deficient buccal bone wall compared with non-grafted sites [71]. Mardas et al. carried out a well-designed prospective randomised controlled trial in which clinical and histological data were correlated following a comparison of two different grafting materials (Straumann Bone Ceramic^®^ (SBC) and Bio-Oss^®^ deproteinised bovine bone mineral (DBBM)) in alveolar ridge preservation (RA) at extraction sockets. In both groups, a collagen barrier was used to cover the grafting material. Complete soft tissue coverage of the barrier membranes was not attempted. After 8 months, before implant placement, the authors re-evaluated the horizontal and vertical dimensions of the residual ridge and performed a histological analysis.

Although a decrease in the bucco-lingual dimension of the alveolar ridge was observed in both groups, the authors reported that both graft materials equally preserved all the other clinical dimensions of the site and supported new bone formation in post-extraction sockets, thus allowing the staged placement of dental implants at a later date. The authors declared the results of this study to be in agreement with previous controlled studies where similar combinations of bone grafts with resorbable barriers were successfully used for alveolar ridge preservation (RA). However, they cautioned that complete preservation of the pre-extraction ridge dimensions in all sites should not be anticipated, as a small amount of crestal dimensional change can still occur after SA. Histological analysis found both biomaterials to have supported new bone regeneration by the process of osteoconduction at the apical and the middle part of the socket [72].

In a high-quality study of SRs, the XV European Workshop in Periodontology (2019) investigated the techniques of alveolar ridge preservation/bone grafting, immediate early and delayed implant placement, and alveolar bone augmentation at the time of implant placement to reach consensus recommendations for the management of the extraction socket. The researchers graded the level of evidence supporting each consensus statement, and its strength was described using a modification of the GRADE tool. The workshop concluded that the extraction sites with a thick buccal bone (e.g., >1.0 to 1.5 mm) exhibited less post-extraction dimensional changes, and SA was more beneficial in sites exhibiting thin buccal bone (evidence level 2/strength of statement: moderate). Furthermore, the need for simultaneous grafting at the time of delayed implantation was less likely in socket-augmented (SA) sites compared with unassisted socket healing [8] (evidence level 2/strength of statement: moderate). They have further concluded that there was no difference in implant success/failure rates after a minimum period of 12 months of loading between implants placed in SA sites compared with unassisted socket healing sites. (evidence level 2/strength of statement: moderate). The workgroup recommended, based on current evidence, a minimum healing period of 3–4 months before implant placement. Furthermore, they recommended that this period should be extended on the basis of the morphological characteristics of the extraction site, the properties of the biomaterial(s) used, and patient-specific systemic factors [73]. Furthermore, there was little evidence to show which biomaterial or socket augmentation technique was superior at the time.

### 2.5. Comparative Study Analysis of Socket Augmentation

To investigate the possibility of marginal bone loss around implants that have been placed in alveolar ridges after SA, Tabrizi et al. have carried out a prospective cohort study with an observation period of 36 months. The authors have compared the stability of marginal bone around implants placed in augmented sockets with implants placed in non-augmented sites and reported no significant difference, concluding that socket augmentation did not contribute to increased marginal bone loss around implants after 36 months of function [74,75].

More recently, a higher level of evidence from well-conducted systematic reviews has been emerging to demonstrate the clinical effectiveness of SA. A systematic review of randomised control trials (RCT) conducted by Avila-Ortiz investigated the effect of using three different SA techniques of “membrane only”, “graft only”, and “graft with a membrane” in extraction sockets. All socket augmentation procedures were found to be effective in limiting horizontal and vertical ridge alterations in post-extraction sites. Interestingly, the meta-analysis also indicated that the use of barrier membranes alone, without a graft, was effective in improving bone healing in extraction sites [76].

In an SR, Mardas et al. (2015) [21] studied implant outcomes and concluded that alveolar SA procedures could decrease the need for further augmentation procedures during staged implant placement, at a later date, compared with unassisted socket healing. Significantly, the success rate, as well as the marginal bone levels of implants placed in alveolar ridges following SA, was found to be comparable with that of implants placed in untreated sockets. Furthermore, there was no evidence that any one biomaterial or SA (RP) technique (GBR, SA, or socket seal) produced better implant outcomes.

In a well-designed RCT, Barone et al. compared the bone dimensional changes following tooth extraction alone with extractions with SA (using corticocancellous porcine bone and a collagen membrane) and carried out a histomorphometric analysis of the grafted sites in comparison with unassisted extraction-alone sites [77]. Their results showed statistically significant differences between the test and treatment sites, both clinically and histologically. The mean reabsorption of the vertical ridge height in the “extraction only” group was 3.6 mm on the buccal sites and 3.0 mm on the lingual sites compared with 0.7 and 0.4 mm, respectively, in the RP group. The authors concluded that the ridge augmentation (SA) approach (using porcine bone in combination with a collagen membrane) significantly limited the post-extraction ridge resorption compared with extraction alone. Furthermore, the histologic analysis showed significantly higher percentages of trabecular bone (35.5–10.4%) and total mineralised tissue in ridge-augmented sites compared with extraction-alone sites (25.7–9.5%) 7 months after tooth removal [77].

Based on quantitative and qualitative analysis of the results of an SR, Avila-Oritz et al. [76] concluded that SA is an effective approach to mitigate against the dimensional reduction in the alveolar ridge that normally takes place after tooth extraction as compared with unassisted healing and recommended SA in conjunction with minimally traumatic tooth extraction to minimise post-extraction alveolar ridge reduction.

The Osteology Foundation 6th Expert Meeting (2011) concluded that the potential benefit of socket preservation therapies using different protocols was demonstrated, resulting in significantly less vertical and horizontal contraction of the alveolar bone crest [78].

Leventis et al. (2014, 2016, 2018, 2020) reported that grafting of extraction sockets without primary wound closure can be an effective method of preserving the contour and architecture of the alveolar ridge using a minimally invasive tooth removal and SA technique using a novel self-hardening β-tricalcium phosphate bone substitute graft material (Ethoss^®^) [79]. At re-entry for implant placement, bone core biopsies were obtained, and primary implant stability was measured by final seating torque and resonance frequency analysis. Histological and histomorphometric analysis carried out by the authors revealed pronounced bone regeneration (24.4 ± 7.9% new bone) in parallel with the conversion of the grafting material (12.9 ± 7.7% graft material) during SA. They have argued that, although a variety of different bone grafts, including xenografts and allografts, have been shown to be effective in SA, the use of a fully resorbable material such as a β-tricalcium phosphate bone substitute would be biologically preferable as it would be fully replaced with new bone without a significant percentage of graft material remaining [75]. One further advantage of using a self-hardening alloplastic graft material in SA is that a barrier membrane is not indicated.

## 3. Bone Grafting and Guided Bone Regeneration

While graft conversion and new bone regeneration have been shown to be relatively slow (compared with allografts, autografts, and synthetics), xenografts have been used predictably and successfully as bone substitute materials for a wide range of defect regeneration in implantology (e.g., sinus grafting and GBR) with a high quality of strong evidence. Extraction sockets are acute defects with a high potential for spontaneous healing. In SA, as in the case of GBR, biomaterials are used as osteoconductive scaffolds to facilitate new bone formation. Xenografts are osteoconductive scaffolds that are commonly used effectively to support GBR for the treatment of dehiscent peri-implant defects by Schwarz et al. [80]. From a biological perspective, the healing process within an augmented extraction socket protected by a barrier membrane should be biologically no different than that of a GBR graft used for the horizontal or vertical augmentation of a deficient alveolar ridge. It can also be argued that the acute extraction sockets possess stronger regenerative potential compared with atrophic, relatively avascular, and acellular residual alveolar ridges.

Different types of bone substitute materials have different physical biomaterial properties, such as crystalline structure and hardness, which affect their volume stability, reactivity, and remodelling characteristics that may account for the variability of results seen in different studies that have been conducted using different types of biomaterials. It is generally accepted that synthetics and allografts are removed and almost completely replaced by new bone within a few months after placement, whereas xenografts are regarded as non-resorbable scaffolds that allow new bone formation around the graft particles. The latter may provide better load-bearing properties compared with the former grafts [81,82].

Nevertheless, there is no evidence currently to show if one biomaterial is superior to another in SA or, indeed, in GBR procedures used in alveolar ridge grafting. While SA was shown to be effective, some authors have argued that grafts can interfere with normal socket healing, and particles of grafting materials may remain in the extraction socket for more than six months and adversely affect the osseointegration process. In contrast, there is little evidence to show that residual bone graft materials adversely affect the osseointegration of dental implants in SA or, indeed, in other grafted sites, such as the maxillary sinus or GBR defects.

Investigating this concern, in an RCT, Barone et al. [77] have histologically showed significantly higher percentages of trabecular bone (35.5–10.4%) and total mineralised tissue in socket-augmented sites compared with extraction-alone sites (25.7–9.5%) 7 months after tooth removal, demonstrating that the use of xenografts did not compromise new bone regeneration in SA. The authors have reported almost complete incorporation of the cortico-cancellous particles in new bone that created a dense and hard tissue structure in which xenograft bone particles were completely surrounded by newly formed vital bone with no sign of inflammatory response or fibrous encapsulation. Barone et al. have concluded that in SA, the xenograft particles acted as an osteoconductive scaffold that supported new bone formation, which acted similarly to the host bone, providing biological support to dental implants.

Another factor that affects the effectiveness of SA is the technique of tooth extraction. Surgical trauma could cause unnecessary damage to the thin labial socket wall, interfere with blood flow, and could predispose to infection, all of which could accelerate crestal bone loss following tooth extraction. Therefore, a flapless, minimally invasive tooth extraction technique using microsurgical instruments such as a periotome or piezo surgery with an extraction insert would be mandatory for achieving successful SA, which is aimed at preserving hard and soft tissues at an extraction site and ensuring the maximum amount of bone regeneration. The use of a barrier membrane in SA has been somewhat controversial, with some studies showing a definitive advantage and others showing no difference in the outcome of SA. One study has shown that a membrane only was successful in producing SA without graft material [74,83]. In the two case studies presented in this paper, no barrier membranes were used. The current authors’ preference is to avoid using barrier membranes or reflecting periosteal flaps unless there is a substantial loss of the buccal plate, in which case a GBR technique is carried out alongside SA.

Jung et al. have recommended the use of a barrier membrane (GBR) during SA when the buccal bone plate was missing 50% or more. They recommended a healing period of 6 months before the placement of an implant and cited the more invasive surgical approach with prolonged treatment duration as the main disadvantage. In smaller buccal bone socket defects (less than 50% buccal bone missing), socket augmentation without a barrier membrane was recommended with deferral of implant placement 4–6 months after SA.

In a recent systematic review of randomised controlled clinical trials analysing the outcomes of flapless socket grafting, Jambhekar et al. reported that, after a minimum healing period of 12 weeks, sockets filled with synthetic biomaterials had the maximum amount of vital bone (45.53%) and the least amount of remnant graft material (13.67%) compared with xenografts and allografts [84,85]. These results are consistent with histomorphometry results of 50.28% of new bone and 12.27% residual graft reported by Leventis et al. for SA using a novel synthetic bone graft composed of calcium sulphate and 12 weeks after a flapless socket grafting procedure. Well-conducted RCTs are urgently needed to demonstrate the comparative characteristics of different biomaterials used in SA. Although the optimum timing of implant placement after SA is not known, there is a general consensus that implant placement should be deferred up to a period of 16–24 weeks after tooth removal. However, it is not known if such an extended period is necessary or even beneficial. More recently, to accelerate the wound healing process, enhancement of SA healing has been advocated with the use of bioactive substances including Platelet Rich Fibrin (PRF), hyaluronic acid (HA), collagen matrix, bone morphogenic proteins (BMPs). PRF contains platelet derived growth factors, hormones, and bioactive components such as cytokines that have been shown to promote angiogenesis and tissue regeneration during all stages of wound healing (see Section 1.4). In addition, the concentration of leukocytes provided in PRF matrix play a crucial role in tissue healing and regeneration as part of osteoimmunological host response to injury.

Benefits of using autogenous PRF platelet concentrates during SA include reduced healing time, enhanced angiogenesis and bone regeneration, socket sealing by fibrin matrix, antibacterial effect, reduced post extraction pain and infection.

In summary, a large number of RCTs and CCTs have demonstrated the substantial benefits of applying SA in sockets immediately after tooth extraction. The cases presented in Figure 6 confirms the benefits of socket preservation. This is consistent with the results of the studies reviewed in this paper. In the current author’s experience, the main benefits of socket augmentation are further enhanced with the application of platelet concentrates (PRF) in conjunction with SA, including: (a) significantly less alveolar/socket wall reduction compared with the conventional technique of unassisted healing; (b) improved bone quality and density as assessed histologically and radiologically; (c) faster soft tissue healing; (d) reduced pain, swelling and lower incidence of alveolitis.

## 4. Conclusions

Alveolar bone atrophy after tooth extraction presents significant challenges when replacing a lost tooth with an implant. Bone loss occurs rapidly during the first 3 months of the post-extraction period and could result in aesthetic, anatomical, and functional problems. There is substantial evidence to show that socket augmentation (SA), at the time of tooth extraction, is an effective therapy in limiting horizontal and vertical ridge resorption compared with unassisted healing. The case study presented in this paper further demonstrated the effectiveness of socket augmentation in preventing dimensional changes from occurring in a tooth socket after extraction. Nevertheless, complete preservation of pre-extraction socket dimensions may not always be achievable at every site, as numerous factors such as the extraction socket morphology, anatomy, thickness of labial socket wall, soft tissue phenotype, and trauma during extraction could adversely affect the dimensional changes and the process of SA.

Socket augmentation is strongly indicated to increase mineralisation and to preserve alveolar ridge morphology where immediate or early implant placement with adequate primary stability is not achievable due to the presence of a large bone defect, damaged socket walls, or unfavourable soft tissue condition. SA has been shown to reduce the need for GBR or bone grafting at early/delayed implant sites. Furthermore, the beneficial effect of SA is also particularly pronounced in the aesthetic zone when the labial socket wall is thin (<2 mm) and more likely to be lost. Ridge preservation (SA) is also indicated when tooth replacement is planned in close proximity of the maxillary sinus or a vital structure such as the neurovascular bundle where the availability of minimum residual bone height may be critical.

To be most effective, SA should be performed in conjunction with minimally traumatic tooth extraction techniques using special instrumentation such as a periotome or piezo surgery (with extraction inserts) to minimise socket wall damage. A flapless extraction technique avoiding periosteal release may be crucial in limiting the dimensional changes occurring to the residual alveolar ridge. There is accumulating good-quality evidence to show the significant bio-enhancement role of platelet concentrates (PRF) when used SA. Currently, there is insufficient evidence to show if one biomaterial or technique is superior to another in SA or if the use of a barrier membrane is beneficial. Further RCTs are needed urgently to further demonstrate the application of SA when managing tooth loss.

## Figures and Tables

**Table 1:** Strategies for management of tooth loss with dental implants (Jonker et al., 2021) [28].

Strategy for Management of Tooth Loss	Immediate Implant Placement in Fresh Extraction Sites	Socket Augmentation (with or without PRF) (+/−Connective Tissue Graft)
**Criteria 1**	Tooth socket intact with >2 mm labial wall thickness	Tooth socket with >50% missing labial wall height
**Criteria 2**	Primary stability is possible in optimum 3D prosthodontically driven implant trajectory	Tooth socket with <1 mm labial wall thickness
**Criteria 3**	Thick soft tissue biotype	Poor soft tissue biotype (e.g., thin/non-keratinised/mobile mucosa), unfavourable smile line

**Table 2:** Factors affecting clinical decision making when considering SA and timing of implant placement (adapted from the XV European Workshop in Periodontology Consensus).

	Factors That Affect Socket Augmentation Treatment
1	Presence of infection (e.g., large cystic lesion)
2	Inability to achieve primary stability in the restoratively driven position
3	Presence of a damaged alveolus (including thin buccal socket wall)
4	Periodontal phenotype
5	Aesthetic demands
6	Systemic conditions

**Table 3:** Randomized clinical trial studies on bone regeneration and soft tissue healing after tooth extraction using PRF [30,40,41,42,43,44,45,46].

Study	Design	Patient Numbers	Teeth Extracted/Region	PRF Type	RPM (RCF [xg]) and Centrifuge Time	Bone Regeneration	Soft Tissue Healing
Ahmed et al. [30]	Parallel RCT	54	Not reported	L-PRF	3000 rpm for 10 min	Radiographic analysis (bone height reduction, crest to tip of root taking adjoining tooth as a guide) after 16 weeks	Wound healing index, resulted in spontaneous healing
Asmael et al. [46]	Split mouth RCT	20	All regions	PRF	3000 rpm for 10 min	Not reported	Percentage of epithelization after 1 week 52.7% and 51.3%, the Landry wound healing index, resulted in spontaneous healing
Giudice et al. [40]	Split mouth RCT	40	All regions	A-PRF+	2700 rpm for 18 min	Not reported	Wound healing index, resulted in spontaneous healing
Marenzi et al. [45]	Split mouth RCT	26	Canines, premolars, and molars	L-PRF	2700 rpm for 12 min	Not reported	Wound healing index, resulted in spontaneous healing
Mourão et al. [41]	Parallel RCT	32	Molars and premolars	L-PRF	3000 rpm for 12 min	Not reported	Wound healing index, resulted in spontaneous healing
Sharma et al. [42]	Split mouth RCT	30	Not reported	PRF	3000 rpm for 10 min	Digital panoramic radiographs after 16 weeks	The Landry healing index, resulted in spontaneous healing
Srinivas et al. [43]	CCT split mouth	30	Maxilla and mandible	L-PRF	3000 rpm for 10 min	CBCT(bone density 24 h, *p* < 0.001	Wound healing index, resulted in spontaneous healing
Ustaoğlu et al. [44]	Parallel RCT	57	Single rooted tooth	L-PRF	2700 rpm for 12 min	Not reported	The Landry healing index, resulted in spontaneous healing

**Table 4:** The optimum socket augmentation technique and criteria.

**Step 1**	**Assess alveolar ridge condition and morphology clinically and radiologically** Check buccal plate condition; Soft tissue biotype (e.g., thick, thin, mobile, or keratinised); Condition of adjacent teeth/periodontal condition; Smile line.
**Step 2**	**Extract using minimally invasive technique and clean and irrigate the extraction socket** Minimally invasive extraction using periotomes and piezo surgery (with an extraction tip) to avoid damage to thin extraction socket walls; Division of multi-rooted teeth before attempting to remove each root using minimally invasive techniques; Degranulation of the socket wall and complete removal of any soft tissue remnants.
**Step 3**	**Augment and suture** Minimal or no reflection of the periosteum. Preserve interdental papilla and vascular supply; Place a particulate graft material and gently condense. Consider soft tissue correction if the soft tissue biotype is unfavourable; Use of a barrier membrane, connective tissue graft, or substitute (e.g., mucograft seal Geistlich^®^) if required to confine the graft material within the socket; Optionally use of PRF fibrin (double layer) Flapless closure using a mattress suture to allow semi-open healing.

## Data Availability

Not applicable.
